# COMPARISON OF CLINICAL BIOMARKER AND DNAM-DERIVED BIOLOGICAL AGE MEASURES IN AN ELECTIVE SURGERY COHORT

**DOI:** 10.1093/geroni/igad104.0375

**Published:** 2023-12-21

**Authors:** Daniel Parker, Shelley McDonald, Kathryn Porter Starr, Dan Blazer, III, E Shelley Hwang, Allan Kirk, Sandhya Lagoo-Deenadayalan

**Affiliations:** Duke University School of Medicine, Durham, North Carolina, United States; Geriatrics, Durham, North Carolina, United States; Durham VA Health Care System, Durham, North Carolina, United States; Surgery, Durham, North Carolina, United States; Surgery, Durham, North Carolina, United States; Surgery, Durham, North Carolina, United States; Surgery, Durham, North Carolina, United States

## Abstract

Older adults face greater post-operative morbidity due to decreasing biological aging (BA)-associated physical resilience. BA measures may quantify these vulnerabilities. Common BA quantification approaches include measures derived from clinical biomarkers and whole blood DNA methylation. The Duke Surgery One Thousand Patient Cohort is a prospective biospecimen and data repository of patients undergoing elective surgery. Using a subset of this cohort (n=119; 61±13 years of age; 50% female; 21% African American), we sought to determine: 1) the inter-relationship of BA measures; 2) associations of race, sex, BMI, and surgery type with BA; and 3) associations of BA with American Society of Anesthesia (ASA) Classification Score . Clinical biomarker BA measures included Klemera-Doubal method (KDM), homeostatic dysregulation (HD), and PhenoAge. DNAm measures included the DNAm PhenoAge, Hannum Age, and GrimAge. We calculated BA advancement (BAA) as BA minus Chronological Age (CA). We calculated Pearson correlation coefficients and fitted regression models, adjusting for CA, sex, race, and BMI. Overall, participants were biologically older than their CA. Male sex and African American race were associated with greater BAA by most measures . Associations of surgery type with BAA were consistent across measures . Greater BAA by most measures was associated with a higher ASA Score, reflecting higher baseline surgical risk. In conclusion, BA measures using different quantitative approaches showed anticipated associations with demographic factors, surgery type, and surgical risk. Ongoing work will identify BA measures that predict adverse surgical outcomes, and that could be used to identify patients for perioperative prehabilitation.

